# In vivo transduction of ETV2 improves cardiac function and induces vascular regeneration following myocardial infarction

**DOI:** 10.1038/s12276-019-0206-6

**Published:** 2019-02-12

**Authors:** Sunghun Lee, Dong Hun Lee, Bong-Woo Park, Riyoun Kim, Anh Duc Hoang, Sang-Keun Woo, Wenjun Xiong, Yong Jin Lee, Kiwon Ban, Hun-Jun Park

**Affiliations:** 1 Department of Biomedical Sciences, City University of Hong Kong, Kowloon Tong, Hong Kong; 2 Department of Pediatrics, Children’s Heart Research and Outcomes Center, Emory University School of Medicine, Atlanta, GA USA; 3 Department of Medical Life Science, College of Medicine, The Catholic University of Korea, Seoul, Republic of Korea; 4 Division of Cardiology, Department of Internal Medicine, Seoul St. Mary’s Hospital, The Catholic University of Korea, Seoul, Republic of Korea; 5 Division of RI-Convergence Research, Korea Institute of Radiological and Medical Sciences, Seoul, Republic of Korea

**Keywords:** Regeneration, Stem-cell research

## Abstract

Vascular regeneration in ischemic hearts has been considered a target for new therapeutic strategies. It has been reported that ETV2 is essential for vascular development, injury-induced neovascularization and direct cell reprogramming of non-endothelial cells into endothelial cells. Thus, the objective of this study was to explore the therapeutic potential of ETV2 in murine models of myocardial infarction in vivo. Direct myocardial delivery of lentiviral ETV2 into rodents undergoing myocardial infarction dramatically upregulated the expression of markers for angiogenesis as well as anti-fibrosis and anti-inflammatory factors in vivo. Consistent with these findings, echocardiography showed significantly improved cardiac function in hearts with induced myocardial infarction upon ETV2 injection compared to that in the control virus-injected group as determined by enhanced ejection fraction and fractional shortening. In addition, ETV2-injected hearts were protected against massive fibrosis with a remarkable increase in capillary density. Interestingly, major fractions of capillaries were stained positive for ETV2. In addition, ECs infected with ETV2 showed enhanced proliferation, suggesting a direct role of ETV2 in vascular regeneration in diseased hearts. Furthermore, culture media from ETV2-overexpressing cardiac fibroblasts promoted endothelial cell migration based on scratch assay. Importantly, intramyocardial injection of the adeno-associated virus form of ETV2 into rat hearts with induced myocardial infarction designed for clinical applicability consistently resulted in significant augmentation of cardiac function. We provide compelling evidence that ETV2 has a robust effect on vascular regeneration and enhanced cardiac repair after myocardial infarction, highlighting a potential therapeutic function of ETV2 as an efficient means to treat failing hearts.

## Cardiovascular disease: New hope for healing the heart

A gene therapy strategy that stimulates cardiovascular repair could improve recovery for heart attack patients. Heart attacks inflict severe damage on the heart and blood vessels, tissues with limited capacity for self-repair. Researchers led by Kiwon Ban of the City University of Hong Kong and Hun-Jun Park of the Catholic University of Korea, Seoul, have now demonstrated that a gene responsible for cardiovascular development can also efficiently stimulate heart repair. They used viruses to deliver the gene into a mouse model of heart attack, and showed that treated heart tissues exhibited strong recovery relative to untreated controls. The treatment reduced scar tissue formation and promoted proliferation of the cells lining blood vessels and blood vessel formation, measurably improving heart function. This approach could lay the groundwork for treating a common potentially fatal event.

## Introduction

Ischemic heart disease (IHD) is one of the most devastating diseases, afflicting ~7.4 million people worldwide^1^. Therefore, effective treatments are urgently needed. In particular, myocardial infarction (MI), the most serious clinical manifestation of IHD, is caused by arterial coronary occlusion (i.e., ischemia), resulting in massive loss of cardiomyocytes and vasculatures^2^. High mortality and morbidity from IHD are mainly ascribed to the limited ability of proliferation of adult cardiomyocytes (CMs) and insufficient coronary vasculature in infarcted hearts^3,4^. Thus, therapeutic strategies have extensively focused on ensuring that enough number of CMs are wired with functionally perfused vasculature in the infarcted heart by manipulating extrinsic or intrinsic signals to promote survival or cell cycle progression of CMs and neovascularization^5,6^.

Since angiogenesis is critical for organogenesis and tissue repair, considerable attempts have been made to treat IHD by promoting myocardial angiogenesis. In particular, pro-angiogenic factors such as VEGF, FGF, and HGF have the focus, among other options, due to their strong ability to induce vessel formation. Administration of VEGF, FGF, or HGF as naked DNA plasmids, viruses, or proteins improved cardiac function and myocardial angiogenesis in animal studies^7–12^. Furthermore, a recent study reported that rapid and transient treatment using VEGF had beneficial effects on MI^13^. After administering modified VEGF RNA into the infarcted heart, functional vessel formation, and myocardial function were enhanced, resulting in an improved long-term survival rate^13^. In addition, findings from a zebrafish study demonstrated that defective angiogenic signaling limited CM proliferation post heart injury, further suggesting neovascularization as an effective therapeutic strategy to treat injured hearts^14^. Despite encouraging experimental reports from numerous preclinical studies, clinical trials with pro-angiogenic factors have generated mixed results^15^. One possible explanation for such discrepancy between animal studies and clinical outcomes might lie in the limited ability of pro-angiogenic factors to induce a diffuse angiogenic program. Since VEGF, HGF, and FGF are ligands for specific signaling pathways, delivery of these factors would be insufficient to fully initiate the process. However, previous studies have demonstrated that some transcription factors, including MYOD, PAX6, and OCT4, can trigger the generation of specific cell lineages in developing embryos or fully differentiated somatic cells by regulating a wide range of lineage-specific genes and acting as a master regulator^16–19^. Thus, identifying potential endothelial master regulator(s) and investigating their functional roles in the vascular system would be of significant importance to developing potent and specific therapeutic vehicles for treating IHD.

ETV2 (also known as ER71) has an essential role in cardiovascular system development^20,21^. In agreement with preferential expression of ETV2 in the hemoangiogenic mesoderm and vascular structures in developing embryos^20–22^, *Etv2*^*-/-*^ mice die in utero due to complete blockage of vascular endothelial cell (EC) and hematopoietic cell development. Mechanistically, ETV2 can interact with other transcription factors to directly activate the expression of genes controlling cardiovascular development and function, indicating potent function of ETV2 in mediating embryonic vessel development^23–26^. Furthermore, ETV2 plays a critical role in postnatal angiogenesis as evidenced by defective new vessel formation in endothelial *Etv2* conditional knockout mice in response to ischemic injury^27^. Recently, it was also reported that ETV2 alone can directly reprogram terminally differentiated somatic skin fibroblasts into functional ECs^28^. Taken altogether, these results strongly support the notion that ETV2 can function as a master regulator of vascular development and regeneration^29^.

Accordingly, the objective of the present study was to examine the therapeutic potential of ETV2 in a murine model of MI. We found that the delivery of lentiviral ETV2 into MI hearts leads to a significant improvement in cardiac repair, including enhanced cardiac function and vessel formation. Regarding mechanisms involved, we identified that ETV2 promoted angiogenesis by directly regulating EC proliferation and indirectly secreting pro-angiogenic factors. Furthermore, injecting an adeno-associated virus (AAV) form of ETV2 had substantial effects on the recovery of cardiac function in rat MI hearts. These results reveal a novel and potent function of ETV2 in mediating cardiac repair and suggest ETV2 as a new therapeutic vehicle for treating patients with heart failure.

## Materials and methods

### Animals

All animal studies were approved by the Institutional Animal Care and Use Committees of the Catholic University of Korea, Korea Institute of Radiological and Biomedical Sciences (Korea).

### Lentiviral ETV2 production

Lentiviral particle production was performed as previously described^27^. In brief, HEK/293T cells transfected with pCSII-EF1α-ETV2-IRES-VENUS (pCSII-EF1α-IRES-VENUS or pCSII-EF1α-FLK1), pCAG-HIVgp and pCMV-VSV-G-RSV-Rev (4:3:1) using the Calcium Phosphate method were incubated for 48 h, and the supernatant was harvested and followed by a PEG-mediated concentration step. The virus titer was determined by a qPCR Lentivirus Titration (Titer) Kit (Abm, Canada). For virus injections into the mouse heart, the infectious unit (IFU) was ~1 × 10^8^/ml.

### Adeno-associated viral ETV2 production

Recombinant Adeno-associated virus (AAV) 9 vectors were produced as previously described^30^. Briefly, AAV vectors, rep2/cap9 packaging plasmids, and adenoviral helper plasmids were mixed with polyethylenimine and added to HEK293T cells (Thermo Scientific). At 72 h post transfection, supernatant and cells were harvested separately for AAV9 preparation. Viruses in the supernatant were precipitated (mixed with 8.5% w/v PEG-6000 and 0.4 M NaCl for 2 h at 4 °C), centrifuged at 7,000 g for 10 min, and resuspended in a virus buffer (150 mM NaCl and 20 mM Tris, pH 8.0). Once the viruses were in the cells, the cell pellet was resuspended in the virus buffer, followed by 3 cycles of freeze-thawing, and dounce homogenization. Cell debris were pelleted at 5000 × *g* for 20 min, and the supernatant was run on an iodixanol gradient. Recovered AAV vectors were washed three times with PBS using Amicon 100K columns (EMD Millipore). Protein gels were run to determine virus titers and purity. Viruses were diluted to various concentrations to test infection, and a concentration of approximately 2 × 10^12^ genome copies (GC)/ml was used for rescue experiments.

### Myocardial infarction model

Animal models of myocardial infarction were created as previously described^31–33^. Briefly, C57BL/6 mice (25–30 g, male, Orient Bio, Korea) or Fisher 344 rats (180–200 g, male, Orient Bio, Korea) were anesthetized with 2.0% isoflurane inhalation. The mouse or rat was intubated via the trachea with an intravenous catheter. They were then mechanically ventilated with medical grade oxygen. After shaving the chest, left thoracotomy was performed. Myocardial infarction was induced by permanently ligating the left anterior descending (LAD) artery. Either the lentivirus or AAV form of ETV2 virus and their control virus (5.6 × 10^6^ IFU per mouse or 2 × 10^12^ GC/ml per rat) were intramyocardially injected at two different sites at the border zone of the myocardium immediately after LAD ligation.

### Echocardiography

Echocardiography was performed to assess functional improvement for injured cardiac tissues^31–33^. The animals were lightly anesthetized with inhaled isoflurane, and physiological data were recorded using a transthoracic echocardiography system equipped with a 15 MHz L15-7io linear transducer (Affiniti 50G, Philips). Different echocardiograms were performed on 7 or 10 mice in each group 1, 2, 4, and 8 weeks after injecting ETV2 virus or control virus into the site of infarction. The echocardiographer was blind to group allocation throughout the experiment. Ejection fraction (EF) and fractional shortening (FS) as indexes of LV systolic function were calculated with the following equations:

$${\mathrm{EF}}\left( {\mathrm{\% }} \right){\mathrm{ = }}\left[ {\left( {{\mathrm{LVEFDD3 - LVESD3}}} \right){\mathrm{/LVEDD}}} \right]\times 100$$

$${\mathrm{FS}}\left( {\mathrm{\% }} \right){\mathrm{ = }}\left[ {\left( {{\mathrm{LVEDD - LVESD}}} \right){\mathrm{/LVEDD}}} \right]\times 100$$

### Immunohistochemistry

Immunohistochemistry was performed as described previously^31–33^. Briefly, heart tissue sections were permeabilized with PBS containing 0.5% Triton X-100 for 15 min at room temperature. Tissue sections were then blocked with 1% BSA in PBS for 60 min at room temperature and incubated with primary antibodies at 4 ℃ overnight. On the next day, samples were washed three times with PBS and incubated with secondary antibodies for 1h at room temperature. DAPI solution (VectaShield) was added for nuclear staining. The primary antibodies used in this study included mouse anti-Vimentin (Millipore; 1:100), rabbit anti-α smooth muscle actin (Abcam; 1:100), anti-cardiac Troponin T (Thermo; 1:100), rabbit anti-CD31 (Abcam; 1:100), mouse anti-CD68 (Abcam; 1:200), rabbit anti-CD206 (Abcam; 1:200), mouse anti-α sarcomeric actinin (Sigma; 1:100), and mouse anti-GFP (Thermo; 1:100). Secondary antibodies used in this study included either anti-mouse/-rabbit IgG Alexa Fluor 488 (Invitrogen; 1:500) or anti-mouse/-rabbit IgG Alexa Fluor 568 (Invitrogen; 1:500). Imaging of heart sections was performed using a Laser Scanning Microscope LSM 880 NLO with Airyscan (Zeiss).

### Quantitative real-time RT-PCR

Total RNAs were extracted by adding 0.5 mL of TRIzol reagent (Life Technologies) to cells on a plate as described in the manufacturer’s instructions. One microgram of RNA was subjected to cDNA synthesis with SuperScriptTM Reverse Transcriptase IV and random primers (Invitrogen). SYBR® Green PCR Master Mix (Applied Biosystems) was used to detect the accumulation of PCR products during cycling with ABI Real-time PCR StepOne Plus (Applied Biosystems). Real-time reverse transcription– polymerase chain reaction (RT-PCR) was carried out in triplicate in three independent experiments. Oligonucleotide primers were designed using real-time RT-PCR sequence detection software v2.3 (Applied Biosystems). Primer sequences are listed in Table S1. Fold differences in the expression level of each gene were calculated for each treatment group using CT values normalized to transcription levels of the housekeeping gene, 18S rRNA or GAPDH according to the manufacturer’s instructions.

### Measurement of fibrosis

Masson’s Trichrome (Sigma) staining was performed to determine the fibrotic area as described previously^31^. Briefly, three frozen slides from each group were fixed in Bouin’s solution for 15 min at 56 °C. After incubation in Weigert’s Iron hematoxylin solution for 5 min at room temperature, slides were stained with Biebrich Scarlet-acid Fuchsin solution for 2 min at room temperature. Samples were then incubated with Aniline Blue for 5 min and 1% acetic acid for 2 min at room temperature. Slides were extensively flushed between each staining. Collagen fibers were stained blue while the myocardium was stained red. The area of fibrosis, in percent, of the left ventricular wall area was quantified using the ImageJ program with basic addons.

### Capillary density

At the time of sacrifice, hearts were perfused with rhodamine-conjugated isolectin B4 from *Griffonia simplicifolia* for 15 min at room temperature. After that, hearts were fixed in 4% paraformaldehyde overnight before embedding in an OCT compound (Thermo Scientific) with dry ice. Transverse cryosection (10 µm) of the fixed tissue was performed using the HM525 NX Cryostat (Thermo Scientific) from the apex to the superior border. These sections were stored in −80 °C before use. The number of capillaries was counted in five random microscopic fields using a fluorescence microscope (Nikon) and expressed as the number of capillaries per square millimeter tissue.

### Isolation of rat neonatal cardiomyocytes, cardiac fibroblasts, and lentiviral transduction

Neonatal rat ventricular cardiomyocytes (NRVMs) and cardiac fibroblasts were isolated from 1- to 2-day-old rats as described previously^34^. Briefly, only ventricles (from the apex to the midline) of neonatal hearts were minced and digested with trypsin (Worthington) at 4 °C overnight. On the next day, tissue pieces were dissociated into single cells with collagenase type II (Worthington) followed by centrifugation at 100 × *g* for 10 min. Cell pellets were suspended with warm culture media and incubated on uncoated plates to save cardiac fibroblasts. Unattached NRVMs were transferred and plated on fibronectin (BD Biosciences)-coated plates. Lentiviral control or ETV2 particles (MOI: 4) were incubated with neonatal myocytes or cardiac fibroblasts for 4–5 days and then subjected to gene expression analysis.

### Scratch assay

Mouse endothelial cell line MS1 (2.8 × 10^4^/well) was cultured overnight to reach a confluent layer in a well separated by an insert (Culture–Insert 2 Well, ibidi GmbH, Germany). Subsequently, the insert was removed to generate a cell-free gap. Then, 1 ml of DMEM medium with 1% FBS + 8% culture supernatants harvested from cardiac fibroblasts infected with either ETV2 or control was added into each well. Images were taken at 0 and 14 h after incubation using a phase-contrast microscope. The area of the cell-free gap was calculated with the ImageJ program.

### BrdU cell proliferation assay

HUVECs transfected with pCSII-EF1α-ETV2-IRES-VENUS or pCSII-EF1α-IRES-VENUS were incubated with BrdU (10 µM) for 3 h and subjected to BrdU staining (APC BrdU Flow Kit, BD Pharmingen) according to the manufacturer’s instructions.

### ^18^F-FDG cardiac PET imaging

Fludeoxyglucose F 18 (^18^F-FDG) uptake in MI hearts was evaluated by using a small animal PET scanner (Inveon^TM^; Siemens Preclinical Solutions). Anesthetized mice or rats were given an intravenous dose of 7.4–11.1 MBq or 14.3–15.9 MBq of ^18^F-FDG (^18^F-fluoro-2-deoxyglucose), respectively. ^18^F-FDG PET imaging was performed for 20 min 1 h after injection. PET emission data were acquired with three spans and 79 ring differences through a 350–650 keV energy window and 3.43 ns timing window. Acquired three-dimensional emission list-mode data were reconstructed for the PET image using the two-dimensional ordered subset expectation maximization (OSEM 2D) algorithm with four iterations. The PET images were then visualized and analyzed using Siemens Inveon Research Workplace (IRW) software (Siemens Preclinical Solutions).

### Image analysis

^18^F-FDG cardiac PET images were processed by rotation and cropping to show only the heart area. Cardiac PET images were converted into the DICOM format to be read by other analysis software. The cardiac PET images of the DICOM format were reoriented to the short-axis, horizontal long axis (HLA), and vertical long axis (VLA) views. Cardiac PET images were then generated as a polar map by using Clinical QGS software (Cedars QGS 2008, Syngo, Siemens). The polar map was segmented to 20 regions to calculate the uptake of ^18^F-FDG in each segmented region. Statistical analysis was performed to compare values of ^18^F-FDG uptake between groups.

### Statistical analysis

All data are shown as the mean ± standard error of the mean (SEM). Statistical differences between the two groups were tested with two-tailed Student’s *t*-tests. Values of *p* < 0.05 were considered statistically significant. All statistical analyses were conducted using SPSS 23.0 (SPSS Inc., Chicago, IL, USA).

## Results

### In vivo transduction of ETV2 into MI-induced hearts

**Fig. 1:**
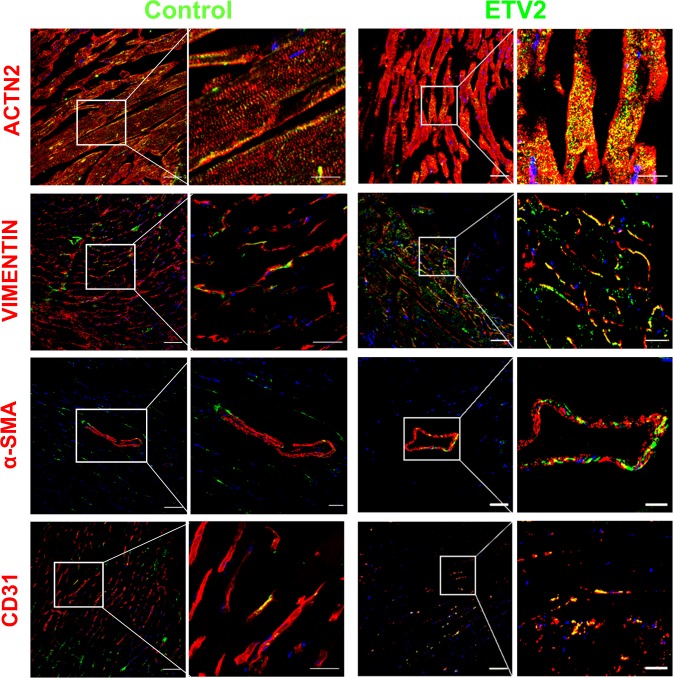
In vivo transduction of ETV2 into MI hearts. Representative confocal immunofluorescent images of sectioned heart tissues harvested 3 days after injection of control or ETV2-Ires-VENUS lentivirus into MI hearts. Anti-ACTN2, VIMENTIN, α-SMA, or CD31 in red and DAPI for nuclei in blue. Scale bars: 50 µm for merged and 20 µm for enlargement (far right panels)

To investigate the applicability of ETV2 as a therapeutic agent for IHD, we employed a murine model of MI induced by permanent ligation of the LAD artery^31–33^. To confirm whether lentiviral particles of ETV2 were effectively delivered into MI hearts, control lentivirus/*Gfp* or lentiviral *Etv2-ires-Venus/Gfp* (herein, ETV2; Figure S1) were intramyocardially injected into mouse hearts. Three days post injection, heart tissues receiving either control or ETV2 lentivirus were excised and subjected to immunohistochemical analysis using anti-GFP antibody and anti-ACTN2, VIMENTIN, -SMA, and CD31 antibodies specific for CMs, endothelial cells (ECs), fibroblasts (FBs), and smooth muscle cells (SMCs), respectively. As shown in Fig. 1, we detected almost equally abundant GFP signals from the four major cell types in the MI hearts: CMs, FBs, SMCs, and ECs (Fig. 1). These histological analysis results clearly indicate successful infection and expression of intramyocardially delivered ETV2 lentivirus into MI hearts.

### Multiple factors involved in angiogenesis, anti-fibrosis, and anti-inflammation are upregulated following ETV2 transduction in MI hearts

**Fig. 2:**
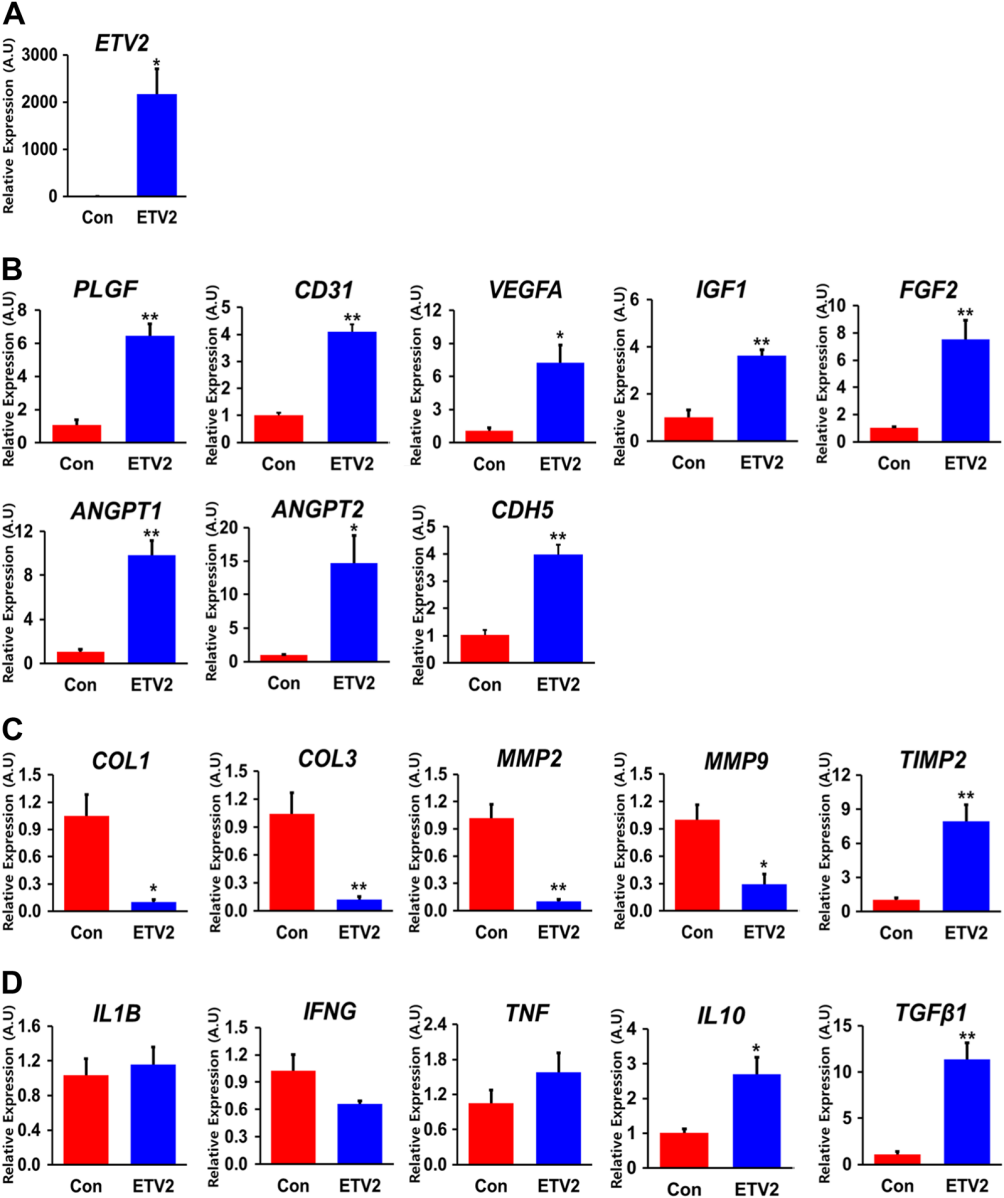
Delivery of lentiviral ETV2 into MI hearts enhances the expression of pro-angiogenic genes but decreases the expression of genes involved in fibrosis and inflammation. Heart tissues harvested 7 days after injection of lentiviral particles of ETV2-Ires-VENUS or control into MI hearts were subjected to qRT-PCR analyses. **a** ETV2 gene, **b** angiogenesis, **c** fibrosis, and **d** inflammation. The *y*-axis represents relative mRNA expression of target genes to GAPDH. A.U. indicates arbitrary units. *p < 0.05 and **p < 0.01 compared to control group, *n* = 3

To explore the effects of ETV2 overexpression on MI hearts, we first performed gene expression analyses using mouse heart tissues harvested on day 7 after the injection of lentiviral particles with either ETV2 or control virus. qRT-PCR results showed that intramyocardially injected ETV2 significantly upregulated the expression levels of ETV2 (>2000 times) (Fig. 2a). Expression levels of prominent angiogenesis-related genes such as VEGFA, FGF2, and angiopoietin 1 & 2 (ANGPT1/2) were significantly upregulated in ETV2-transduced hearts (Fig. 2b), while cardiac fibrosis markers, such as collagen type 1 (COL1), collagen type 3 (COL3), and matrix metalloprotease 2/9 (MMP2/9), were substantially downregulated. However, tissue inhibitors of metalloproteinase 2 (TIMP2), an anti-fibrosis-related gene, was upregulated. These results suggest an anti-fibrotic effect of ETV2 (Fig. 2c). Of interest, while expression levels of pro-inflammatory factors including interleukin 1 beta (IL1B) and interferon gamma (IFNG) were not significantly different between the two groups (ETV2 and control), expression levels of anti-inflammatory factors such as interleukin 10 (IL10) and transforming growth factor beta 1 (TGFβ1) were significantly higher in the ETV2 injected group than those in the control group (Fig. 2d). Additionally, to further verify the anti-inflammatory effects of ETV2, immunohistochemistry was performed with CD68 and CD206 antibodies using the MI heart tissues obtained 1 week after injection with control or ETV2 lentivirus. The immunohistochemistry results demonstrated that the number of cells positive for CD68, a marker for monocyte lineage cells, was not significantly different between the two groups (Figure S2A). On the other hand, the number of positive cells for CD206, a type 2 macrophage maker, was substantially higher in the ETV2 virus injection group compared to the control virus-injected group. These results also support the notion that overexpression of ETV2 induced anti-inflammatory effects in MI hearts (Figure S2B). Collectively, these results indicate that overexpression of ETV2 in MI hearts increased multiple biological factors associated with vascularization, anti-fibrosis, and anti-inflammation.

### ETV2 improves cardiac function and reduces scar formation after MI

**Fig. 3:**
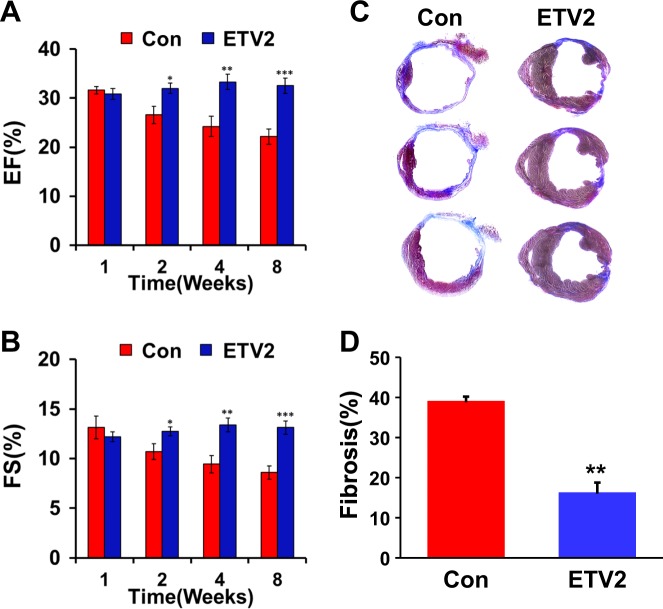
Overexpression of ETV2 significantly improves cardiac function and reduces cardiac fibrosis in a mouse model of myocardial infarction. Mice undergoing MI were injected with lentiviral particles of ETV2-Ires-VENUS or control followed by echocardiography analysis. **a**, **b** Ejection fraction (EF) and fractional shortening (FS) were significantly higher in the ETV2-injected group compared to those in control virus-injected group, *n* = 7–10 for each group. **c**, **d** Representative images showing collagen deposition (blue) in the left ventricle of mouse hearts by Masson’s Trichrome staining. Bar graphs summarizing the percentage of fibrosis in the left ventricle of mouse hearts. For each heart, three randomly selected sections from the apex to ligated area were measured, *n* = 6 for each group. *p < 0.05, **p < 0.01, and ***p < 0.001 compared to control

Given that immediate regulation of gene expression was favorable for recovery from MI upon ETV2 injection, we reasoned that lentiviral delivery of ETV2 into MI hearts could improve cardiac function. To determine the therapeutic effects of ETV2 overexpression in MI hearts, we performed echocardiography on a weekly basis to measure cardiac remodeling and function in mice that received lentiviral particles of ETV2 or control. Echocardiography results demonstrated that delivery of ETV2 into MI hearts significantly improved cardiac function compared to the control virus-injected group as determined by ejection fraction (EF) (32.5% ± 1.5% in ETV2 group vs. 22.2% ± 1.6% in control group, *n* = 7–10; *p* < 0.01) and fractional shortening (FS) (13.1% ± 0.7% in ETV2 group vs. 8.6% ± 0.7% in control, *n* = 7–10; *p* < 0.01) (Fig. 3a, b). Importantly, Masson’s trichrome staining of cardiac tissues harvested at 8 weeks also confirmed that the ETV2-injected group showed significantly reduced cardiac fibrosis compared to the control group (16.3 ± 2.4% in ETV2 vs. 39.1 ± 1.1% in control, *n* = 3; *p* < 0.05) (Fig. 3c, d). Analyses of myocardial perfusion (^99m^Tc-MIBI SPECT/CT) and glucose metabolism (^18^F-FDG PET) further validated that lenti-ETV2 injection into MI hearts improved cardiac function (Figure S3). Taken together, these results from a series of independent assessments clearly suggest the therapeutic effects of ETV2 on MI hearts.

### Effects of ETV2 on vascular regeneration in MI hearts

**Fig. 4:**
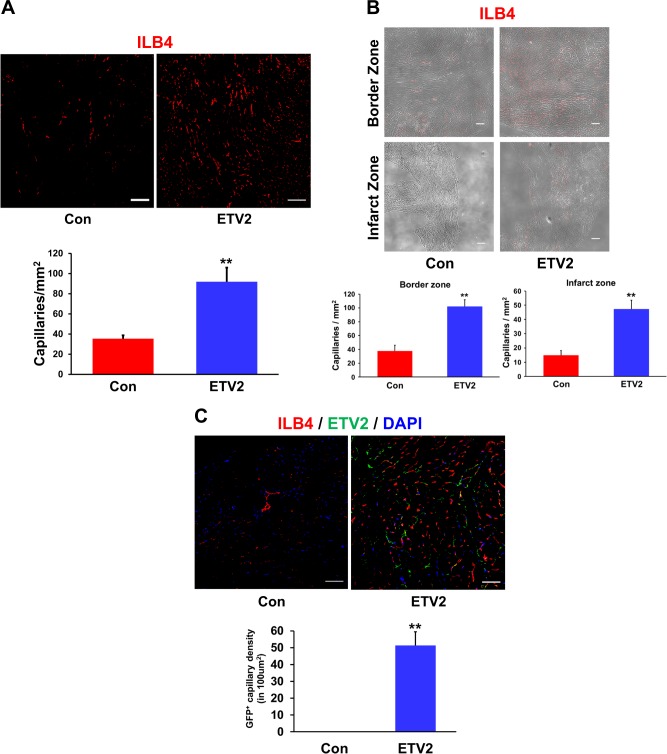
ETV2 overexpression increases capillary density in MI hearts. MI hearts of mice receiving lentiviral particles of ETV2-Ires-VENUS or VENUS were perfused with rhodamine conjugated IB4 to visualize vessels. They were subjected to immunohistochemical staining with anti-GFP antibody, IB4 (red), ETV2 (green), and DAPI for nuclei (blue). **a** Representative images of capillaries 8 weeks after MI (upper panels) and summarized quantification (lower panels), scale bars: 100 µm. For quantification, the number of capillaries in five randomly selected fields in each heart was counted, *n* = 5–7. **b** Representative images of capillaries (upper panels) and summarized quantification (lower panels) in the border zone and infarct zone in individual test groups, scale bars: 100 µm. *n* = 5. **c** A significant number of vessels were double-positive for both IB4 (red) and ETV2 (green) in ETV2-injected heart tissues. Representative confocal images of double-positive capillaries (upper panels) and summarized quantification (lower panels) in heart tissue harvested 8 weeks after ETV2 injection, scale bars: 50 µm. *p < 0.05, **p < 0.01 compared to control group

Since ETV2 was found to be a strong inducer of neovascularization that could significantly contribute to cardiac regeneration following MI, we evaluated the effects of ETV2 on vascular regeneration in MI hearts. To this end, we perfused isolectin B4 (IB4) conjugated with a red fluorescent dye into the heart to visualize the vessels prior to tissue harvest 8 weeks after ETV2 injection. Fluorescent image assessment demonstrated that the number of capillaries in ETV2-injected hearts was significantly higher than in the control group (Fig. 4a; 91.9 ± 13.9 in ETV2 group vs. 35.3 ± 3.4 in control group, *n* = 5–7; *p < 0.05). Of interest, the number of capillaries in both the border zone and infarcted area in the ETV2 virus-treated group were significantly higher than those areas in the control virus-treated group (91.9 ± 13.9 in ETV2 group vs. 35.3 ± 3.4 in control group, *n* = 5–7; *p *<* 0.05) (Fig. 4b).

Next, to determine the potential and magnitude of the contribution of ETV2 to vascular regeneration in MI hearts, we traced the signal from ETV2-GFP within cardiac tissues. Confocal microscopic analyses revealed that a significantly higher number of vessels double-positive for both IB4 and GFP signals (i.e., ETV2) were observed in the region of the infarct in the ETV2 virus-injected heart tissues compared to those in control virus-injected group 8 weeks after virus injection (Fig. 4c and Figure S4A). Heart tissues harvested at week 17 after virus injection (Figure S4B) showed similar results. Of note, GFP signals (i.e., ETV2) were not detected from ACTN-2-positive CMs or VIMENTIN-positive CFs 8 weeks post infection (Figure S5). Taken together, these results suggest that ETV2 can directly contribute to augment vascular regeneration in vivo in ischemic hearts.

### ETV2 promotes migration and proliferation of endothelial cells

**Fig. 5:**
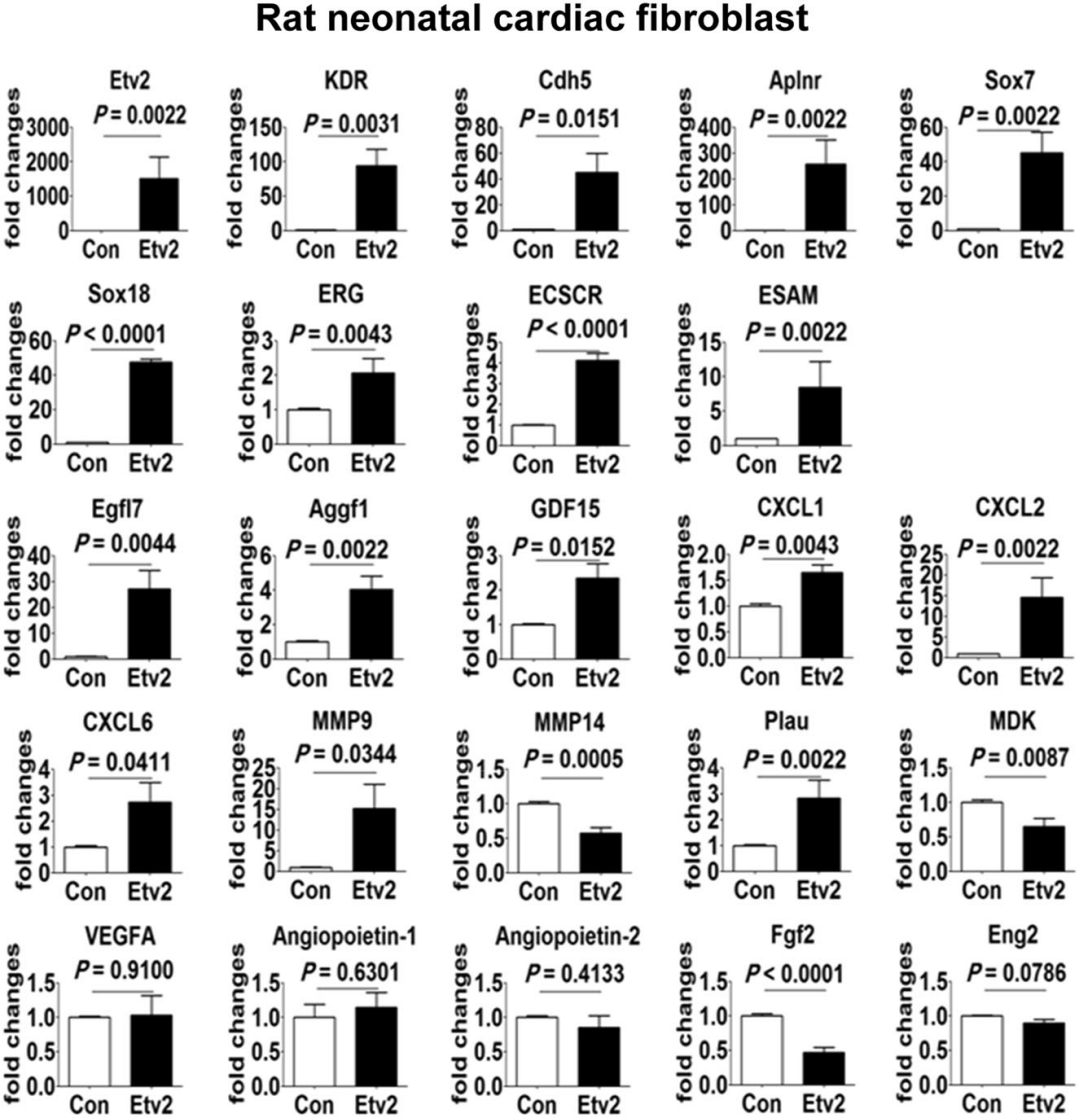
Gene expression analysis in cardiac fibroblasts infected with ETV2. Rat neonatal cardiac fibroblasts infected with lentiviral ETV2 were subjected to gene expression analysis. The *y*-axis represents fold change in mRNA expression compared to that in control virus-infected cells. *p*-values were calculated compared to the control group, *n* = 3

ETV2-mediated enhancement of vascular regeneration in MI heart would be ascribed to a proliferative advantage of ECs infected with a high level of lentiviral ETV2. Alternatively, non-ECs infected with ETV2 might be able to promote myocardial angiogenesis by secreting pro-angiogenic factors or through direct conversion into ECs. Accordingly, to obtain insights into ETV2-mediated cardiac recovery with significantly enhanced vessel formation, we infected CMs and CFs isolated from neonatal rat hearts with ETV2 lentiviral or control particles in vitro. As expected, significantly higher levels of ETV2 expression was evident in ETV2-infected CMs or CFs compared to those of control viral particle infected cells. During transduction with ETV2 for 4–5 days, we did not observe any significant morphological changes of CMs or CFs into EC-like cells (data not shown), suggesting that trans-differentiation or direct conversion with ETV2 is unlikely to be a major contributor to augmented vascularization in MI hearts (see Discussion). Next, we determined the expression levels of genes related to vasculogenesis or angiogenesis. As shown in Fig. 5, CFs infected with ETV2 showed significant upregulation of key endothelial genes such as KDR, CDH5, SOX7, SOX18, ERG, APLNR, ESAM, and ECSCR. The expression levels of angiogenesis genes such as EGFL7, MMP9, PLAU, AGGF1, and GDF15 were also upregulated upon ETV2 transduction. The expression of anti-angiogenic MDK was decreased, whereas the expression levels of pro-angiogenic cytokines, such as CXCL1, CXCL2, and CXCL6, were increased in ETV2-infected CFs (Fig. 5). However, delivery of ETV2 into CMs failed to induce the expression of these genes, except SOX7, SOX18 and EGFL7 (Figure S4). Expressions of well-known angiogenic cytokines including VEGFA, VEGFB, FGF, and ANG2 in the absence or presence of ETV2 overexpression in both CMs and CFs were comparable (Fig. 5 and Figure S6). Collectively, these results suggest that CFs infected with lentiviral ETV2 but not CMs promoted the functional recovery of infarcted hearts by regulating angiogenesis partly through secreted factor(s).

**Fig. 6:**
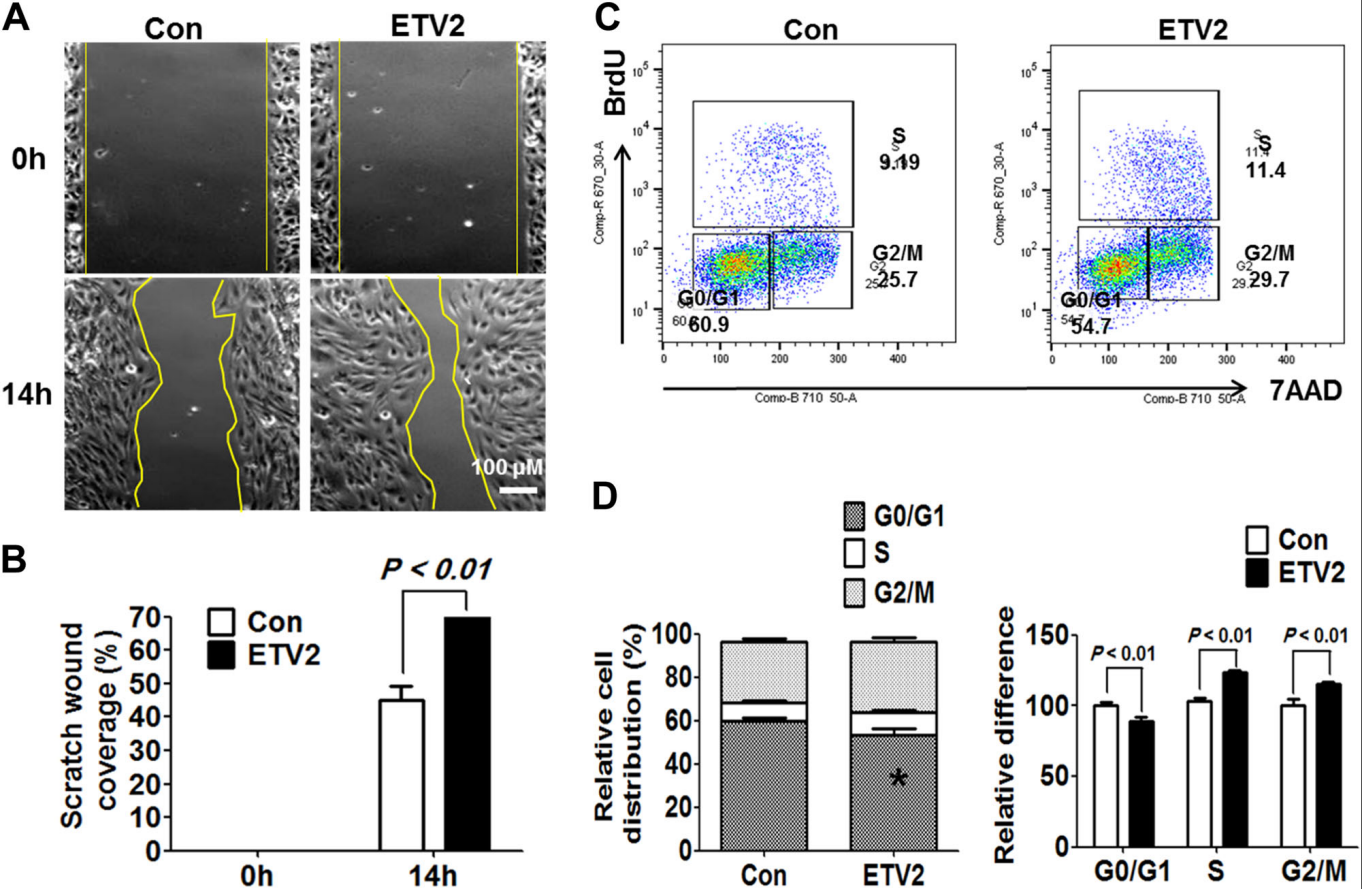
ETV2 promotes migration and proliferation of endothelial cells. **a**, **b** Scratch assay. Culture supernatant harvested from rat neonatal cardiac fibroblasts infected with lentiviral ETV2 or control was incubated with mouse endothelial cells upon ‘scratch’ (see Methods). **a** Representative images under an inverted microscope and **b** quantification of scratch wound coverage**. c**, **d** Increased proliferation of endothelial cells in response to ETV2. HUVECs transfected with control virus or ETV2 lentivirus were incubated with BrdU for 3 h and subjected to FACS-BrdU analysis. **c** Representative flow analysis and **d** quantification

To examine whether CFs infected with ETV2 could indirectly promote angiogenesis through secreted factors, we cultured mouse endothelial cells (MS1 cell line) and subjected them to a scratch assay, an in vitro experiment for cell migration/proliferation as critical steps in angiogenesis. The culture supernatant was then collected from CFs infected with ETV2 or control virus. As shown in Fig. 6 and Figure S7, the culture supernatant harvested from CFs infected with ETV2 showed an enhanced ability to fill the gap compared to that of the control virus-infected group (70.24 ± 0.50% in ETV2 group vs. 45.06 ± 7.09% in control group, *n* = 3; *p < 0.05). To avoid the possibility of EC infection from lentiviral particles present in the culture supernatant, the culture was washed at least three times with PBS 16 h after infection (Fig. 6a, b and Figure S7).

Since ETV2 could infect ECs in MI hearts (Fig. 1), we explored whether ETV2 in ECs was able to promote cell proliferation by BrdU assay. ECs infected with lentiviral ETV2 showed increased proliferation as demonstrated by enhanced portions of S and G2/M phases with decreased G0/G1 upon ETV2 overexpression (Fig. 6c, d). Taken together, these results suggest that enhanced vessel formation in MI hearts upon ETV2 transduction is partly due to enhanced EC proliferation by direct function of ETV2 in EC and angiogenic factors secreted from ETV2-infected CFs.

### Therapeutic applicability of ETV2 with adeno-associated virus for clinical use

**Fig. 7:**
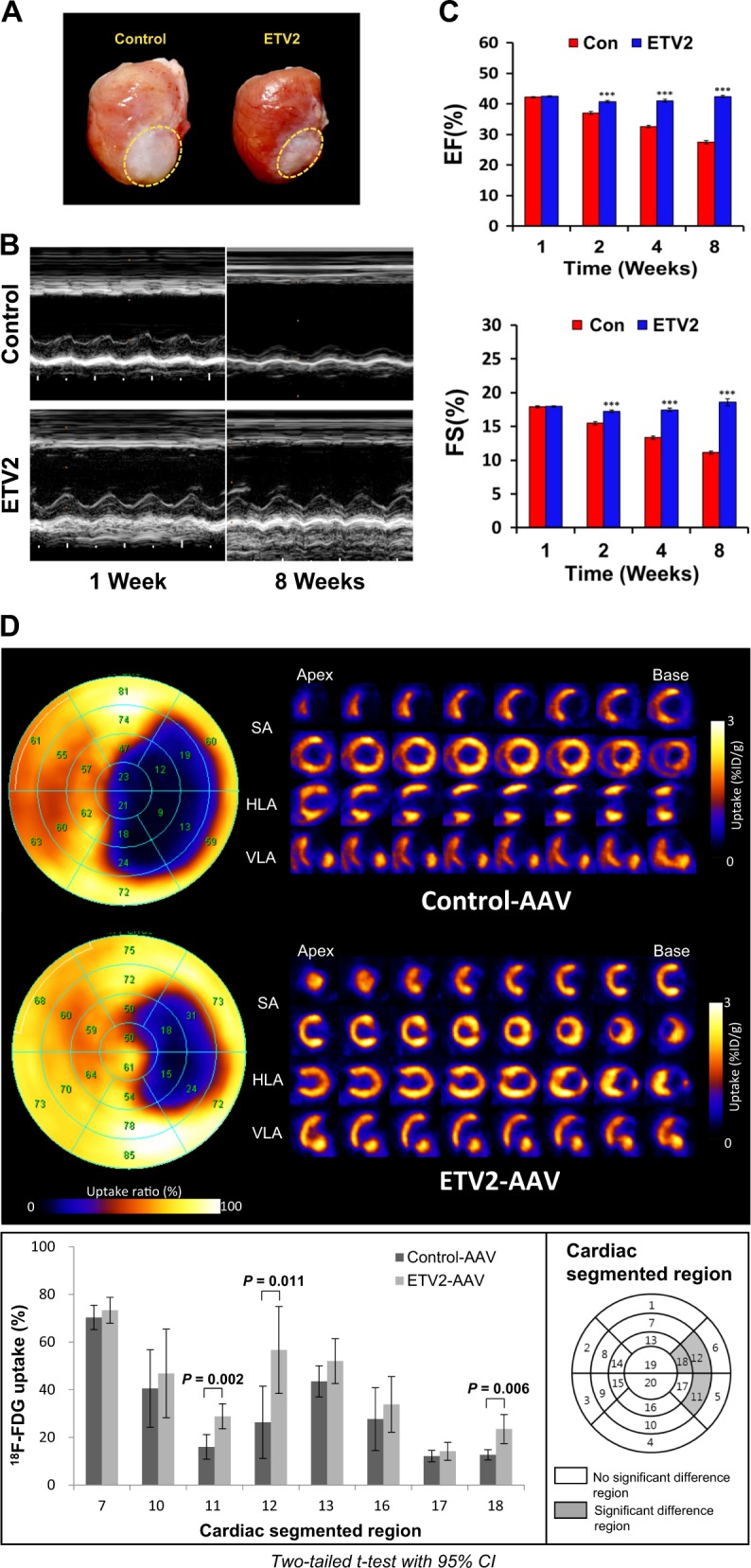
Myocardial injection of AAV9-ETV2 improves cardiac function in a rat model of myocardial infarction. AAV9-ETV2 and control virus were injected into MI hearts of rats followed by image and functional analysis. **a** Optical images of sacrificed hearts 8 weeks after injection. White circle shows the area of fibrotic scar formation. **b** Representative images of echocardiography. **c** Quantitative analysis of EF and FS, ***p < 0.001. **d** Analysis of ^18^F-FDG cardiac uptake PET images with left ventricular polar maps (upper panels). Color on the map represents summed value of ^18^F-FDG uptake in the left ventricle. Cardiac ^18^F-FDG PET image was reoriented to the short-axis (SA), the horizontal long-axis (HLA), and the vertical long-axis (VLA). Quantification of ^18^F-FDG PET image in 20-segment polar map (bottom panels). Scores (%) of ^18^F-FDG uptake in the infarct area showed significant reduction in AAV-ETV2 group compared to that in the control group (*n* = 6, *p* < 0.05)

To further explore the therapeutic applicability of ETV2, we developed therapeutic platform of ETV2 by employing an AAV-9 delivery system^35,36^. Optical images of MI hearts sacrificed 8 weeks after injection of AAV control showed eccentric remodeling and large infarct size in the control group. In contrast, significantly smaller infarct size and less adverse remodeling were observed in AAV-ETV2 treated MI hearts (Fig. 7a). Serial echocardiography showed that both EF (42.4 ± 1.4% in AAV-ETV2 group vs. 27.5 ± 1.3% in control group) and FS (18.6 ± 1.5% in AAV-ETV2 group vs. 11.1 ± 0.6% in control group) were significantly higher in the ETV2-injected group than the control group (*n* = 6, *** *p* < 0.001) 8 weeks post injection (Fig. 7b, c).

For more accurate evaluation of cardiac viability, we additionally performed ^18^F-FDG PET scans 8 weeks after AAV-ETV2 injection. Left ventricular polar maps showed that ^18^F-FDG uptake was significantly higher in the AAV-ETV2-treated group compared to control group (Fig. 7d, upper panels). Of note, we detected a significant difference in ^18^F-FDG uptake between the two groups, particularly in the mid-inferolateral region (segment 11: 28.8 ± 5.2% in AAV-ETV2 group vs. 16.0 ± 5.1% in control group, *p* = 0.002), mid-anterolateral region (segment 12: 56.7 ± 18.3% in AAV-ETV2 group vs. 26.3 ± 15.2% in control group, *p* = 0.011), and apical anterolateral wall (segment 18: 23.8 ± 6.1% in AAV-ETV2 group vs. 12.7 ± 2.2% in control group, *p* = 0.006) (Fig. 7d, lower panels). Moreover, when the infarct regions were analyzed with a cross-sectional view (Figure S8), AAV-ETV2-treated hearts showed substantially higher ^18^F-FDG uptake than control hearts, indicating smaller infarcted areas (^18^F-FDG uptake: 0.91 ± 0.19% injected dose/gram tissue (ID/g) in AAV-ETV2 group vs. 0.52 ± 0.07% ID/g in control group, *n* = 6; *p* = 0.032). Taken altogether, these results indicate that delivery of ETV2 in an AAV-9 into rat MI hearts consistently promoted cardiac function, suggesting that ETV2 has substantial therapeutic potential for treating MI hearts.

## Discussion

Following MI, promoting vascular regeneration is one the most effective options for treating failing hearts. Despite its clinical importance, due to the failure of several clinical trials with pro-angiogenic factors such as VEGF, FGF, and HGF in MI patients, currently available strategies for therapeutic vascular regeneration are very limited. In the present study, we demonstrate the first evidence that in vivo transduction of ETV2 into infarcted hearts by local delivery of lentiviral ETV2 leads to a significant improvement in cardiac repair and enhanced cardiac function accompanied by an increase in vascular regeneration. Furthermore, we show that the AAV form of ETV2 is as efficient as lentiviral ETV2 in a murine model of MI, raising the possibility of using ETV2 as a promising therapeutic agent for the treatment of MI patients.

We found that capillary density in infarcted myocardium was significantly increased following ETV2 transduction in MI hearts. While lentiviral ETV2 efficiently infected four major cell types (CMs, FBs, ECs, and SMCs) in the heart by week 1, the majority of transgenic ETV2 expression was only detected in ECs in capillaries and not in other cell types 8 weeks postinjection. We also found that ECs infected with ETV2 in vitro showed enhanced proliferation, suggesting that ETV2 in ECs directly contributes to vascular regeneration in MI hearts. In agreement with our interpretation, previous findings revealed that ETV2 plays a critical role in vascular development and angiogenesis. Park et al. demonstrated that mice lacking *Etv2* die in utero due to complete absence of vascular and blood development, which was further confirmed by other groups^37,38^. In a subsequent study, they showed that endothelial-specific ablation of *Etv2* leads to impaired vascular regeneration upon ischemic insult^27^. Conversely, a single treatment with lentiviral ETV2 was sufficient to regenerate blood vessels and induce subsequent tissue repair in ischemic mouse hindlimbs^27^. In the current study, we further verified that overexpression of ETV2 enhanced EC proliferation and angiogenesis via the VEGF/FLK1 signaling cascade. Taken together, it is tempting to speculate that ETV2 can activate an angiogenic program in ECs and thus generate more vessels in MI hearts, leading to improvement in myocardial perfusion and cardiac function.

In addition to the direct effects of ETV2 on angiogenesis, recent studies have demonstrated the role of ETV2 in direct conversion of non-ECs (i.e., fibroblasts and skeletal muscle) into EC-like cells, suggesting the possibility that some proportion of newly formed vessels in MI hearts originated from non-ECs through direct conversion upon lentiviral ETV2 injection^28,39–41^. Ginsberg et al. showed that ETV2 in combination with other ETS factors such as FLI1 and ERG contributed to direct conversion of human amniotic cells into functional ECs^39^. Han et al. demonstrated that a pool of five vasculogenic/endothelial transcription factors (FOXO1, ETV2, KLF2, TAL1, and LMO2) can successfully reprogram mouse fibroblasts into ECs^40^. Subsequently, our recent study demonstrated that ETV2 alone was sufficient to directly reprogram cultured human dermal fibroblasts (HDFs) into ECs^28^. A similar finding was reported in zebrafish showing direct conversion of skeletal muscle into ECs by ETV2 overexpression in vivo^41^. In the present study, while we found a significant upregulation of key endothelial genes from CFs upon ETV2 overexpression, we did not detect noticeable morphological changes of ETV2-infected CFs into ECs or EC-like cells, suggesting that direct conversion of non-ECs to ECs by ETV2 is not a major pathway promoting coronary revascularization in the MI heart. Since the analysis was performed in vitro, we could not rule out the possibility that CFs with a high level of ETV2 in vivo can be converted into EC-like cells in response to MI. In a recent study, it was shown that ~30–40% CFs contribute to coronary vessel formation through mesenchymal-to-endothelial transition in the injured heart^42^, suggesting plasticity of CFs particularly after cardiac injury. Therefore, future studies are needed to reveal the ability of ETV2 to mediate direct conversion of CFs into ECs in MI hearts. However, CMs did not show significant changes in EC genes examined following ETV2 transduction in the present study.

Paracrine factors released from ETV2 transduced cells in MI hearts could be another important mechanism for ETV2-mediated cardiac repair. Indeed, the regenerative potential of paracrine factors secreted from different cell types has received great interest in cardiovascular regenerative medicine due to their favorable effects on various cellular functions, including cell proliferation, migration, and survival^43,44^. In this study, we found that culture supernatant harvested from CFs transduced with ETV2 enhanced wound closure compared to that from control virus. Although we have very limited information on what factor(s) might play a major role in this process, gene expression analysis suggested that EGFL7 from ETV2-infected CFs might promote wound closure of ECs considering its role in vessel development and angiogenesis in pathophysiological conditions^45^. In addition, upregulated expression of MMP9 and Plau and pro-angiogenic chemokines, such as CXCL1 and CXCL2^46^, in response to ETV2 in CFs could enhance angiogenesis. Genome-wide transcriptome analysis might help us identify secreted factor(s) from ETV2-transduced CFs or CMs that can promote vascular regeneration in MI hearts. Collectively, we envision that enhanced cardiac function upon ETV2 transduction in MI hearts is due to enhanced angiogenesis via EC proliferation and migration through both direct and indirect pathways.

One major finding in our current study was that ETV2 showed therapeutic applicability in MI hearts. Delivery of AAV ETV2 significantly promoted cardiac function comparable to that of lentiviral ETV2. Considering the relatively low risk of pathogenicity, immune response, and long-term expression of transgenes, AAV vectors have been instrumental in designing gene therapy for clinical use^35,36^. Therefore, our results provide a novel opportunity for ETV2-mediated gene therapy in treating MI hearts. Additionally, delivery of non-viral forms of ETV2, such as modified RNA or endogenous ETV2 gene expression, in specific cell types of hearts using Crispr-Cas9 technology or small molecules might have great benefits for devastating diseases. Since the rapid regeneration of new blood vessels is essential for prolonging the survival of injured myocardium, future studies are warranted to identify detailed mechanisms underlying ETV2-mediated cardiac function recovery in MI hearts. This study may serve as a proof-of-concept approach that ETV2-based treatment can be used as a therapeutic tool to promote neovascularization and cardiac regeneration.

## Supplementary information

Supplementary Materials
