# Author Correction: In vivo transduction of ETV2 improves cardiac function and induces vascular regeneration following myocardial infarction

**DOI:** 10.1038/s12276-019-0271-x

**Published:** 2019-09-04

**Authors:** Sunghun Lee, Dong Hun Lee, Bong-Woo Park, Ri Youn Kim, Anh Duc Hoang, Sang-Keun Woo, Wenjun Xiong, Yong Jin Lee, Kiwon Ban, Hun-Jun Park

**Affiliations:** 1 Department of Biomedical Sciences, City University of Hong Kong, Kowloon Tong, Hong Kong; 2 Department of Pediatrics, Children’s Heart Research and Outcomes Center, Emory University School of Medicine, Atlanta, GA USA; 3 Department of Medical Life Science, College of Medicine, The Catholic University of Korea, Seoul, Republic of Korea; 4 Division of Cardiology, Department of Internal Medicine, Seoul St. Mary’s Hospital, The Catholic University of Korea, Seoul, Republic of Korea; 5 Division of RI-Convergence Research, Korea Institute of Radiological and Medical Sciences, Seoul, Republic of Korea

**Correction to: Experimental & Molecular Medicine**

10.1038/s12276-019-0206-6 published online 12 February 2019

In the original version of this article the author name Riyoun Kim was published incorrectly. This should be Ri Youn Kim.

