# Supplementary material for: In vivo transduction of ETV2 improves cardiac function and induces vascular regeneration following myocardial infarction
Source: Exp Mol Med. 2019 Feb 12;51(2):1–14. doi: 10.1038/s12276-019-0206-6 (PMC6372609; doi:10.1038/s12276-019-0206-6)
Supplement: Supplementary file 1 — Supplementary Materials [file 12276_2019_206_MOESM1_ESM.pdf]

SUPPLEMENTAL MATERIALS

Supplementary methods

^{99m}Tc-MIBI cardiac SPECT/CT

^{99m}Tc-MIBI (Technetium-99m-labelled methoxyisobutyl isonitrile (^{99m}Tc-sestamibi, Cardiolite®); Du Pont) uptake in the infarcted myocardium was evaluated by using a small animal Single-Photon Emission Computed Tomography (SPECT) scanner (Inveon™; Siemens Preclinical Solutions). Mice were anesthetized with 2.0% isoflurane during image acquisition and given an intravenous dose of 111-148 MBq of ^{99m}Tc-MIBI. SPECT imaging was performed for 60 minutes at 3 hours after virus injection. CT images were acquired at an X-ray voltage of 80 kVp and an anode current of 500 µA with an exposure time of 200 msec. SPECT images were acquired with the following parameters, 143–175 keV energy window, 1.0 mm one pinhole collimator, 39 mm axial FOV, 70 mm radial FOV, and 30 seconds for each degree. SPECT images were reconstructed using maximum a posteriori 3D (MAP3D) with point spread function. The SPECT/CT images were visualized and analyzed using Siemens Inveon Research Workplace (IRW) software (Siemens Preclinical Solutions).

Image analysis

^{99m}Tc-MIBI cardiac SPECT images were processed by rotation and cropping to show only the heart area. Cardiac SPECT images were converted into the DICOM format to be read by other analysis software. The cardiac SPECT images of the DICOM format were re-oriented to the short-axis, horizontal long axis (HLA), and vertical long axis (VLA) views. Cardiac SPECT images were then generated as a polar map by using the Clinical QGS software (Cedars QGS 2008, Syngo, Siemens). The polar map was segmented to 20 regions to calculate the uptake of ^{18}F-FDG in each segmented region. Statistical analysis was performed to compare values of ^{18}F-FDG uptake between groups.

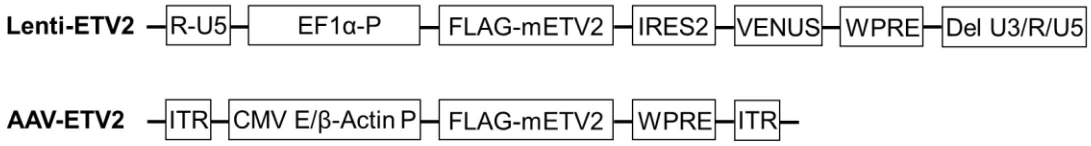

Figure S1. The schemes of vectors used in this study. Lenti virus (Upper) and Adeno associated virus 9 (AAV9; lower).

U: unique, R: repeat, ITR: AAV inverted terminal repeat, WPRE: Woodchuck Hepatitis Virus (WHP) Posttranscriptional Regulatory Element, E: enhancer, P: promoter.

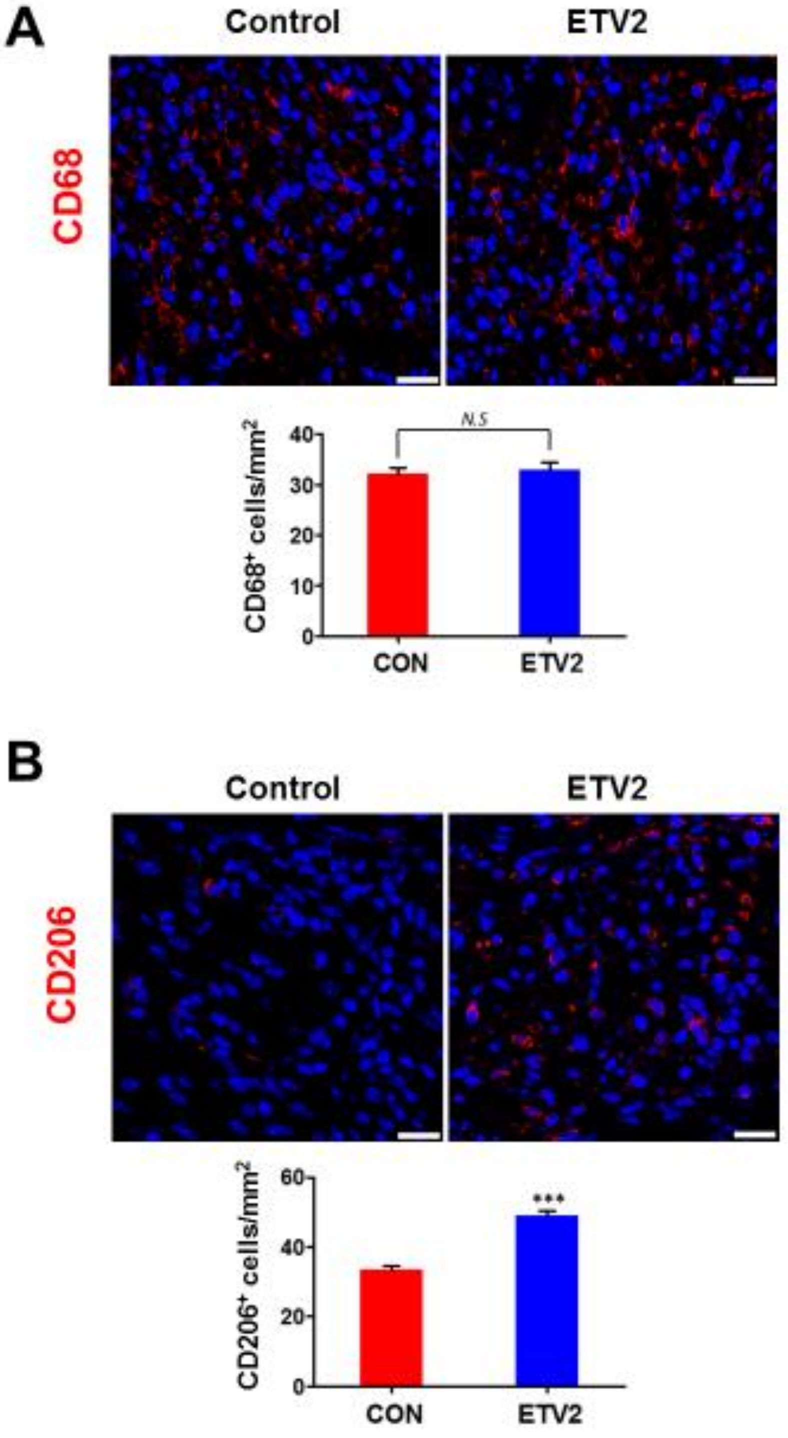

Figure S2. Anti-inflammatory effects of ETV2 in MI hearts. MI hearts of mice receiving control or ETV2 lentiviral particles were subjected to immunohistochemical staining with CD68 and CD206 antibodies (red) and DAPI for nuclei (blue). **(A)** Representative images of CD68 at 1 weeks after MI and their quantification summary. Scale bars: 20 µm. N=3. **(B)** Representative images of CD206 and their quantification summary. Scale bars: 20 µm. n = 3. ***p < 0.001 compared to control group.

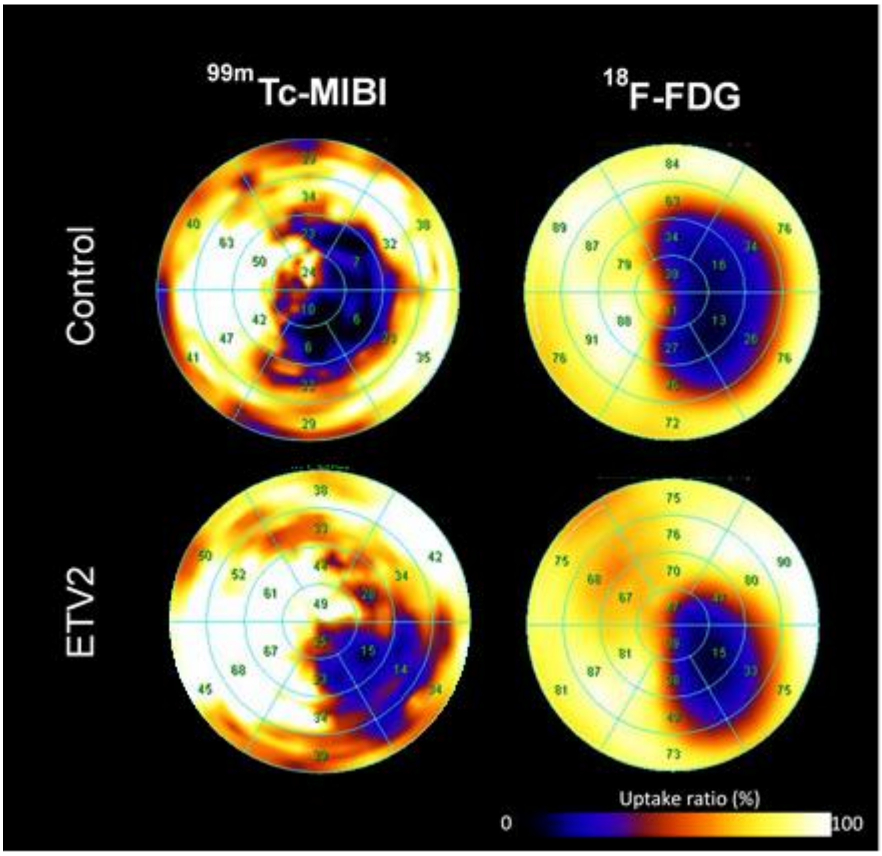

Figure S3. Assessment of myocardial perfusion and glucose metabolism on myocardial infarction-induced hearts. Myocardial perfusion images of SPECT/CT (^{99m}Tc-MIBI) and glucose metabolism image of PET image (^{18}F-FDG) in control (n=3) and ETV2 virus injected mice (n=3) at 8-weeks after injection. All 20 segmented regions were calculated as ^{18}F-FDG uptake ratio. Higher uptake ratio was represented in yellow and low uptake ratio represented in blue or black.

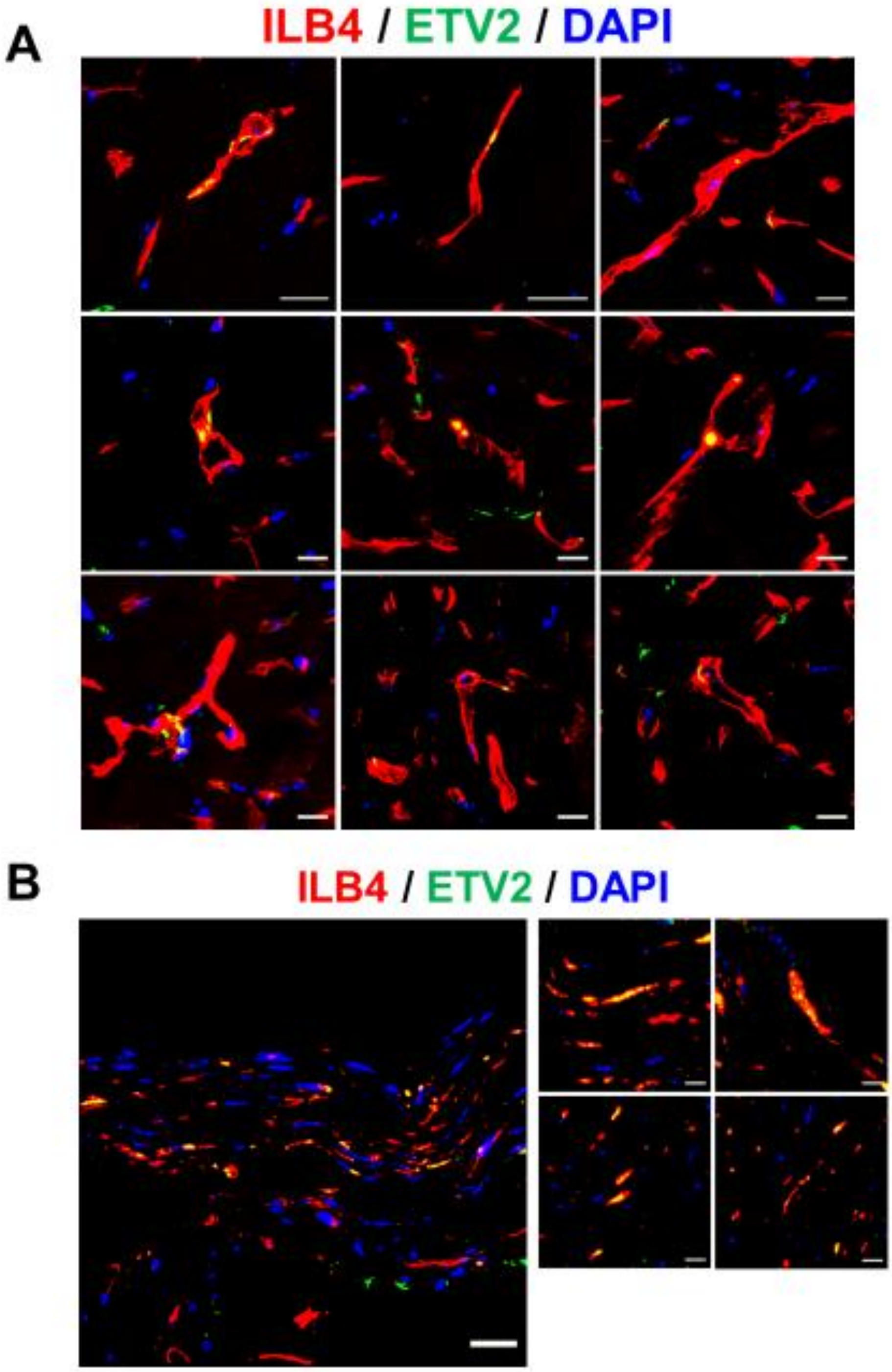

Figure S4. ETV2 overexpression increases capillary density in MI induced hearts. MI hearts of mice receiving lentiviral particles of ETV2-Ires-VENUS were perfused with rhodamine conjugated IB4 to visualize vessels. They were subjected to immunohistochemical staining with anti-GFP antibody, IB4 (red), ETV2 (green), and DAPI for nuclei (blue). **(A)** Enlarged confocal images of double positive capillaries in heart tissues at 8 weeks after injection. Scale bars: 10 μm. **(B)** Confocal images of double positive capillaries in hearts tissue at 17 weeks after injection. Scale bars: 20 μm.

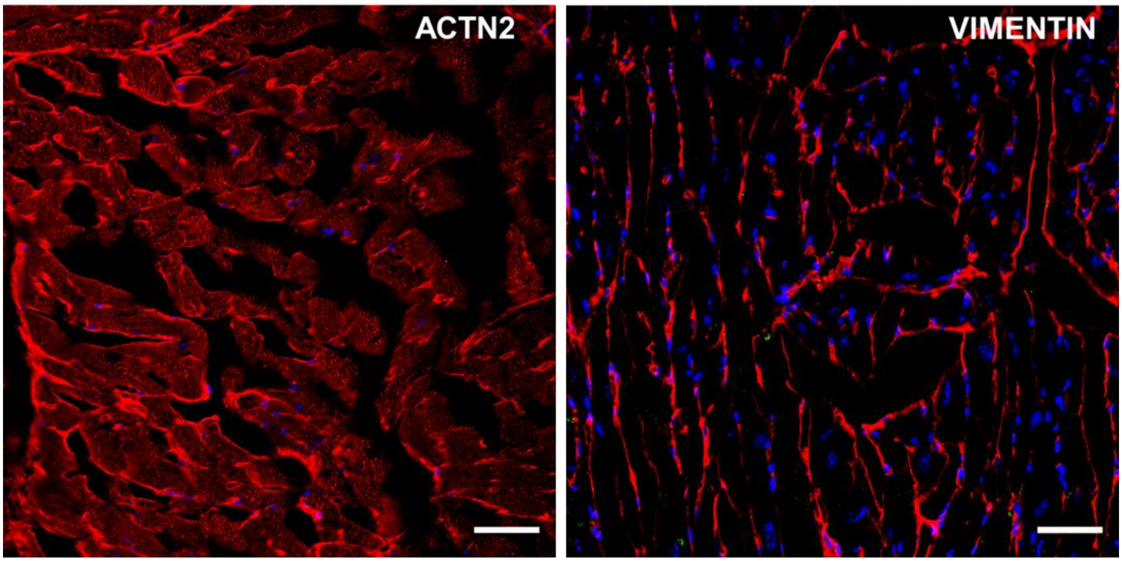

Figure S5. Tracing of GFP positive signals on the mouse hearts at 8-weeks after MI. Representative immunofluorescent images of heart histological sections from ETV2 virus injected mice at 8 weeks from the injection. Heart sections were stained with GFP antibody (green), nuclei in blue and either ACTN2 and Vimentin in red. (scale bars: 50 μm)

Rat neonatal cardiomyocytes

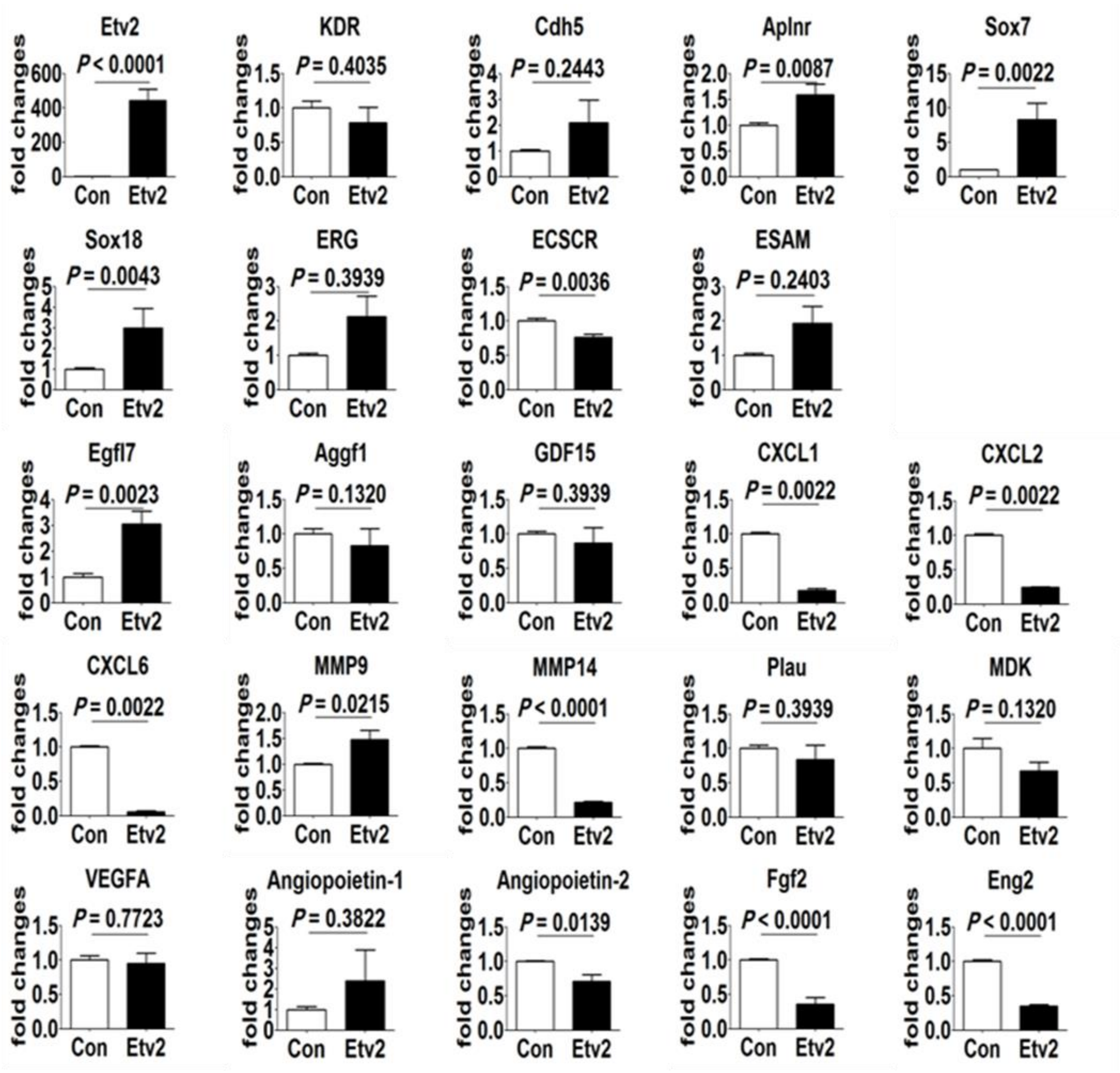

Figure S6. Gene expression analysis in cardiomyocytes infected with ETV2. Neonatal rat cardiomyocytes infected with lentivial ETV2 were subjected to gene expression analysis. The y axis represents mRNA expression of fold change to control virus infected cells as control. P values was calculated compared to control group; n=3.

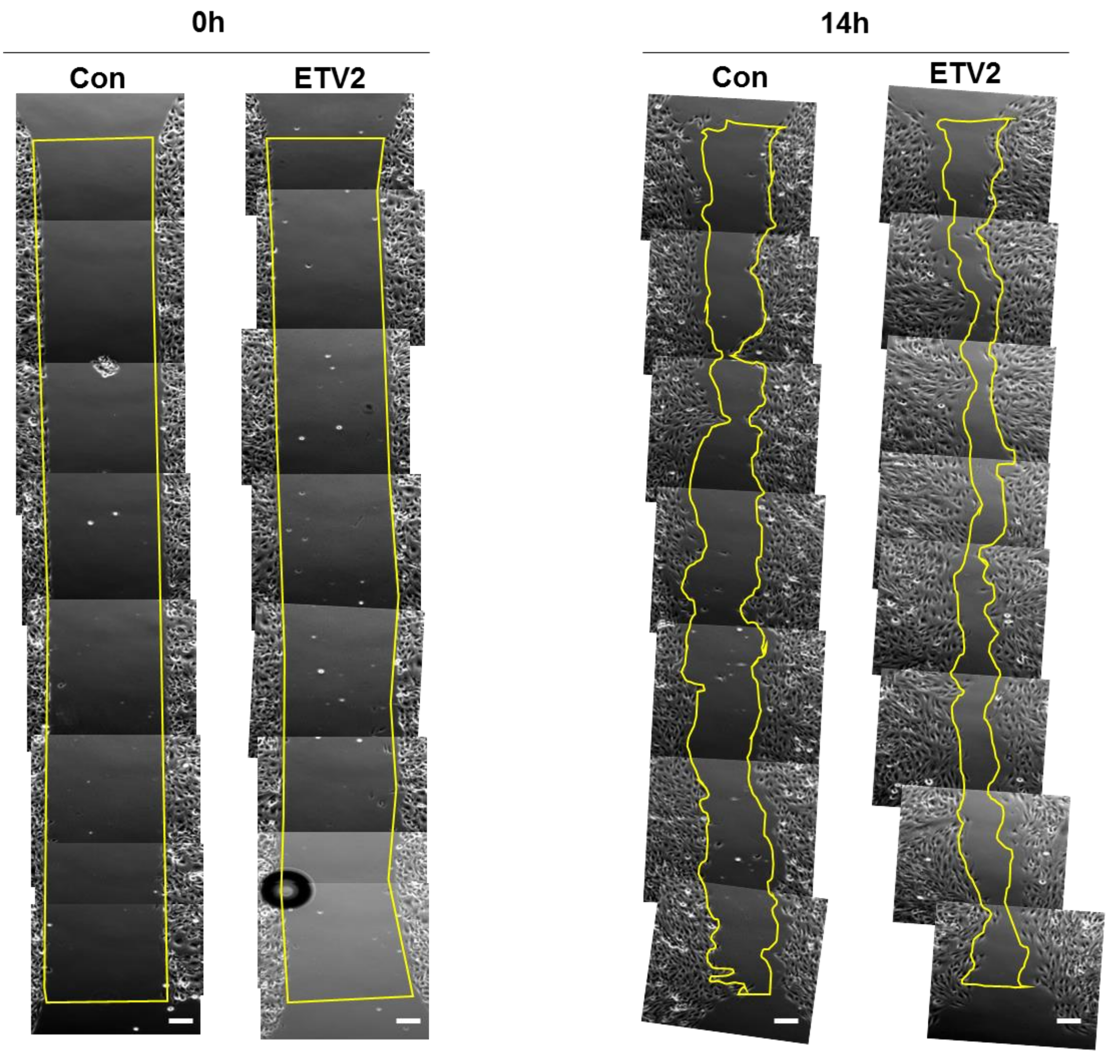

Figure S7. Enhanced gap closure by the supernatant of ETV2-infected cardiac fibroblasts. The culture supernatant harvested from neonatal cardiac fibroblasts infected with lentiviral ETV2 or control was incubated with MS1 cells upon ‘scratch’ (see Methods). Representative limages under an inverted microscope. Left panels: 0 hr, right pannles: 14 hrs after the scratch. Scale bars: 100 μm.

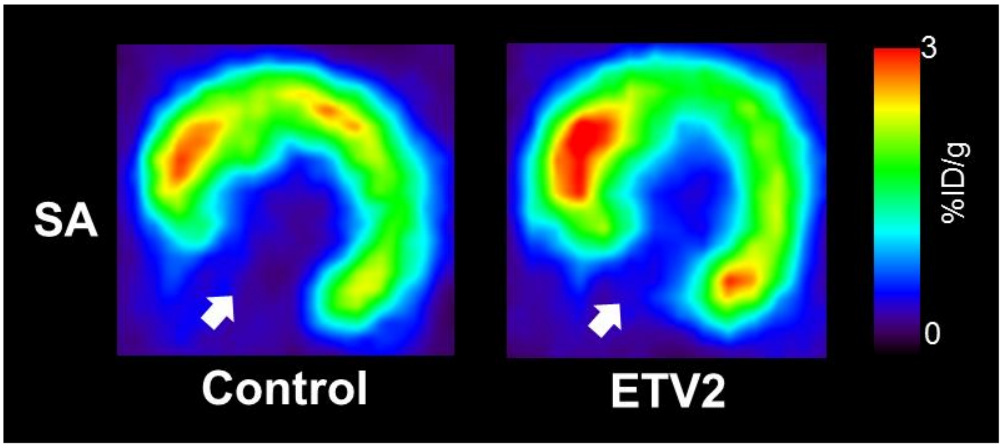

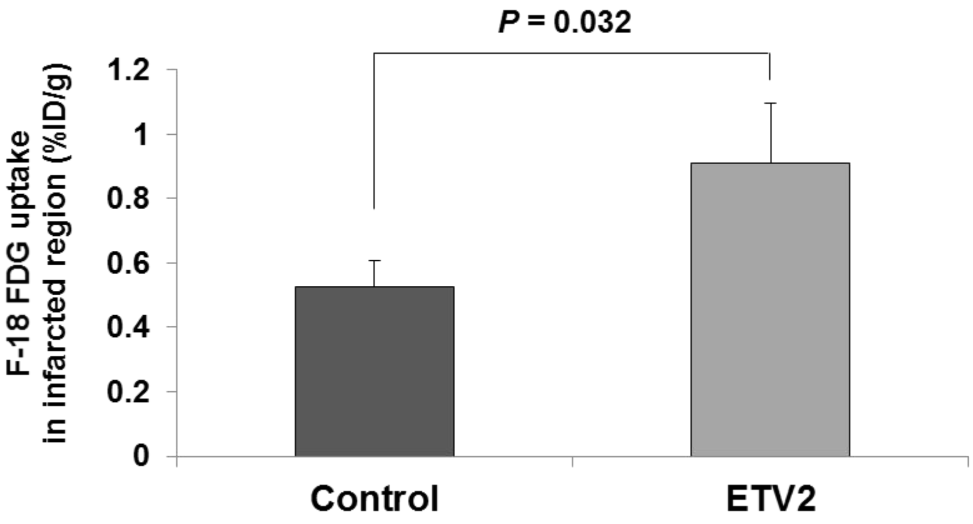

Figure S8. Assessment of 18F-FDG PET images with cross-sectional view in the infarct region. 18F-FDG uptakes of infarcted area (short-axis view) were measured and expressed in percentage of injected dose/gram tissue (%ID/g) (upper panels, representative figures). White arrow indicates infarcted region. Quantification of myocardial 18F-FDG uptakes (lower panel). n=6, p=0.032.

Table S1. The list of primers used for real time RT-PCR.

Target gene	Sequences	
	**Forward (5′-3′)**	**Reverse (5′-3′)**
18S rRNA	CGC GGT TCT ATT TTG TTG GT	AGT CGG CAT CGT TTA TGG TC
ER71	CAG AGT CCA GCA TTC ACC AC	AGG AAT TGC CAC AGC TGA AT
PLGF	CTG TGT GCC GAT AAA GAC AGC	GGT TCC TCA GTC TGT GAG TTT C
CD31	GAG CCC AAT CAC GTT TCA GTT T	TCC TTC CTG CTT CTT GCT AGC T
VEGFa	CAT AGA GAG AAT GAG CTT CCT ACA GC	TGC TTT CTC CGC TCT GAA CAA GG
IGF-1	CTG GAC CAG AGA CCC TTT GC	GGA CGG GGA CTT CTG AGT CTT
FGF-2	GCG ACC CAC ACG TCA AAC TA	CCG TCC ATC TTC CTT CAT AGC
Angpt1	TGC ACT AAA GAA GGT GTT TTG CT	CCG GTG TTG TAT TAC TGT CCA A
Angpt2	CAG CCA CGG TCA ACA ACT C	CTT CTT TAC GGA TAG CAA CCG AG
CDH5	TGG AGA AGT GGC ATC AGT CAA CAG	TCT ACA ATC CCT TGC AGT GTG AG
ColI	TAG AGG CTC TGA AGG TCC CC	CAC CAG CAA TAC CAG GAG CA
ColIII	GTG AAA CTG GTG AAC GTG GC	ATA GGA CCT GGA TGC CCA CT
MMP2	GAT ACC CTC AAG AAG ATG CAG AAG T	ATC TTG GCT TCC GCA TGG T
MMP9	AAA CCC TGT GTG TTC CCG TT	CAG GCT GTA CCC TTG GTC TG
TIMP2	GGA CAC GCT TAG CAT CAC CCA GA	GTC CAT CCA GAG GCA CTC ATC C
IL 1β	TGG TGT GTG ACG TTC CCA TT	CAG CAC GAG GCT TTT TTG TTG
IFNG	GTC CAG CGC CAA GCA TTC	GCT GGA TTC CGG CAA CAG
TNF	CAG CCG ATG GGT TGT ACC TT	GGC AGC CTT GTC CCT TGA
IL10	AAG CAT GGC CCA GAA ATC AA	CGC ATC CTG AGG GTC TTC A
TGFβ1	CTT CAA TAC GTC AGA CAT TCG GG	GTA ACG CCA GGA ATT GTT GCT A
